# Availability and readiness of healthcare facilities and their effects on long-acting modern contraceptive use in Bangladesh: analysis of linked data

**DOI:** 10.1186/s12913-022-08565-3

**Published:** 2022-09-21

**Authors:** Md Nuruzzaman Khan, Shahinoor Akter, M. Mofizul Islam

**Affiliations:** 1 Department of Population Science, Jatiya Kabi Kazi Nazrul Islam University, Mymensingh, 2222 Bangladesh; 2 Gender and Women’s Health, Centre for Health Equity School of Population and Global Health, The University of Melbourne, Melbourne, Victoria 3010 Australia; 3 Department of Public Health, La Trobe University, Melbourne, 3086 Australia

**Keywords:** Modern contraceptives use, contraception, linked data, health facility data, multilevel regression, Bangladesh

## Abstract

**Aim:**

Increasing access to long-acting modern contraceptives (LMAC) is one of the key factors in preventing unintended pregnancy and protecting women’s health rights. However, the availability and accessibility of health facilities and their impacts on LAMC utilisation (implant, intrauterine devices, sterilisation) in low- and middle-income countries is an understudied topic. This study aimed to examine the association between the availability and readiness of health facilities and the use of LAMC in Bangladesh.

**Methods:**

In this survey study, we linked the 2017/18 Bangladesh Demographic and Health Survey data with the 2017 Bangladesh Health Facility Survey data using the administrative-boundary linkage method. Mixed-effect multilevel logistic regressions were conducted. The sample comprised 10,938 married women of 15–49 years age range who were fertile but did not desire a child within 2 years of the date of survey. The outcome variable was the current use of LAMC (yes, no), and the explanatory variables were health facility-, individual-, household- and community-level factors.

**Results:**

Nearly 34% of participants used LAMCs with significant variations across areas in Bangladesh. The average scores of the health facility management and health facility infrastructure were 0.79 and 0.83, respectively. Of the facilities where LAMCs were available, 69% of them were functional and ready to provide LAMCs to the respondents. The increase in scores for the management (adjusted odds ratio (aOR), 1.59; 95% CI, 1.21–2.42) and infrastructure (aOR, 1.44; 95% CI, 1.01–1.69) of health facilities was positively associated with the overall uptake of LAMC. For per unit increase in the availability and readiness scores to provide LAMC at the nearest health facilities, the aORs for women to report using LAMC were 2.16 (95% CI, 1.18–3.21) and 1.74 (95% CI, 1.15–3.20), respectively. A nearly 27% decline in the likelihood of LAMC uptake was observed for every kilometre increase in the average regional-level distance between women’s homes and the nearest health facilities.

**Conclusion:**

The proximity of health facilities and their improved management, infrastructure, and readiness to provide LAMCs to women significantly increase their uptake. Policies and programs should prioritise improving health facility readiness to increase LAMC uptake.

## Introduction

Unintended pregnancy represents around 44% of all pregnancies in low- and middle-income countries (LMICs) [1]. Almost one-third of them occur due to contraceptive failures, primarily because of using short-term modern contraceptives (e.g., pills and condoms) and traditional contraceptives (e.g., withdrawal, rhythm) [2]. In LMICs, unintended pregnancies are responsible for around 55 million unplanned births and 25 million miscarriages in a year [1]. These constitute around 80% burden of pregnancy complications (including severe bleeding and infections) [3], leading to over 75% of the 118,000 maternal deaths each year in LMICs [4]. The underlying reasons are adverse health and behavioural issues following unintended conception, during pregnancy and the post-partum period, including depression, anxiety and lower use of intra-partum, delivery and post-partum care [5–7]. Furthermore, such high occurrences of unintended pregnancies in LMICs are attributed to 138 million abortions annually [1]. Abortion is prohibited in many LMICs, even though having an abortion can save a woman’s life. This prohibition and scarce safe abortion facilities result in unsafe abortions, increasing maternal morbidity and mortality. Also, unwanted pregnancy forces many women to carry their fetuses to term and postpartum and experience the socioeconomic strain of the subsequent births and health risks. Access to a full range of contraceptives and family planning services means that women are more likely to find something that works in their circumstances and increase the likelihood of consistent and correct use. Indeed, contraception use can avert about 60 million healthy lives lost [8] and around 10% of child deaths in LMICs per year [9]. The injectable, female strelization, male strelization, intrauterine devices (IUD) and hormonal implants are the long-acting modern contraception (LAMC) that can significantly reduce these burdens [10]. Accordingly, universal access to sexual and reproductive healthcare services, including increasing use of LAMC, has been targeted in the Sustainable Development Goals (SDGs, Targets 3.7) to be achieved by 2030 [11]. This is also considered an essential pathway to achieving another two SDG targets: reducing maternal (Target 3.1) and child mortality (Target 3.2).

In Bangladesh, an LMIC, around half of the pregnancies are unintended at conception, and over one-third are attributed to contraceptive methods failure [2, 12]. Consequently, the government has focused on increasing contraception coverage, particularly LAMC use [12]. Contraceptives are being provided from all existing healthcare facilities and community clinics, strengthening the traditional approach of providing family planning services and contraception by family planning workers [13]. Targeted programs are also implemented to increase health facilities’ capabilities and readiness to offer a full range of contraceptive methods [12]. The target is to enable couples to access contraceptive methods that meet their needs. A similar approach has been taken in many other LMICs. However, to what extent this endeavour contributes to an increase in LAMC use is an ongoing debate in Bangladesh, as the prevalence of modern contraceptive use has been minimal over the years, along with evidence of rising pregnancies due to contraceptive failures [13]. As a result, policymakers are in a dilemma about the contribution of healthcare facilities to LAMC use and cannot decide whether they should prioritise the health facility over the traditional approach of family planning services at the community level by family planning workers [14]. This dilemma, to some extent, is due to insufficient evidence on how contraception availability in health facilities affect contraceptive use — a lack that also suppresses the true effects of population-level factors [15–18]. Thus far, limited research has been conducted mostly in African countries to examine the effect of health facility-level factors on the overall use of contraception [19–22]. However, those studies did not present a stratified analysis for rural and urban areas, despite substantial variations in the distribution of health facilities and contraception-using patterns in these two settings. The norms of contraceptive use and associated factors for Bangladesh are significantly different from African countries, along with a substantial rural-urban variation [2]. However, previous studies on this topic in Bangladesh considered only the population-level factors [2, 23, 24]. To address these gaps, we conducted this study to examine the effects of availability and accessibility of health facility-level factors on LAMC use in Bangladesh.

## Methods

### Study overview

We analysed data extracted from 2017/18 Bangladesh Demographic and Health Survey (BDHS) and 2017 Bangladesh Health Facility Survey (BHFS). We established linkage between these two survey datasets using the administrative boundary linkage method [25, 26]. The Demographic and Health Survey Programme of the USA designed these surveys, and the National Institute of Population Research and Training conducted the surveys under the supervision of the Ministry of Health and Family Welfare of Bangladesh. Detailed sampling procedures of these surveys can be found in the respective survey reports [27, 28]. Briefly, the 2017/18 BDHS collected nationally representative sample selected by using multistage stratified random sampling methods. At the first stage of sampling, 675 clusters were selected from a list of 293,579 clusters that was created as part of the 2011 National Population Census. A fixed number of 30 households were selected at the second stage from each cluster through probability proportional to the unit size. Of them, data were collected from 19,457 households. There were 20,376 eligible women in the selected households of which 20,127 women were included in the survey with a response rate 98.8%. The 2017 BHFS used a list of 19,811 registered health facilities as a sampling frame generated by the Ministry of Health and Family Welfare. A total of 1600 health facilities were selected from this list, and 1524 health facilities were finally included in the survey.

### Sample

We analysed data from 10,384 women selected from 672 clusters included in the BDHS 2017/18. The criteria used for inclusion in this study were women of reproductive age (15–49), who were fertile but not pregnant or experiencing lactational amenorrhea at the time of the survey and did not desire a child for 2 years prior to the date of the data collection.

### Outcome variable

The outcome variable was LAMC use. The relevant data were collected by asking women, “*Are you currently doing something or using any method to delay or avoid getting pregnant?*” Women who responded “Yes” to this question were then asked, “*Which method are you using?*” and could select their method from the following list: pill, injectable, implant, intrauterine device (IUD), condoms, female sterilisation, male sterilisation, periodic abstinence and withdrawal. A free text option was also provided to report the name of contraception if not included in the list. If a woman reported multiple methods, the method they used most frequently was selected. Responses were coded as “1” for LAMC (i.e., the injectable, implant, IUD, female sterilisation and male sterilisation) and “0” for contraceptive non-use (responded “no” to the first question) or for use of traditional methods (i.e., periodic abstinence or withdrawal). A further classification was made as use or non-use of long-acting reversible methods (injectable, IUD and implants) and parament methods (male sterilisation, female sterilisation). Women who reported condoms or pills as their current method of contraception were excluded, as these are available at local shops and pharmacies in Bangladesh as well as supplied by the family planning staff working at the field level.

### Explanatory variables

The explanatory variables in this study were selected in three stages: at the first stage, we conducted a comprehensive literature search in five databases (PubMed, CINHAL, Web of Science, Embase and Google Scholar) and identified all relevant studies conducted in LMICs, particularly in Bangladesh [15, 16, 18, 23, 24, 29–36]. The variables considered in these selected studies were then summarised, and their availability was then checked with the dataset we analysed in the second stage. The summarized variables that were available in the survey were then considered with the outcome variable of this study and forward regression models were run. Less significant variables (*p* > 0.20) were removed from the list. Furthermore, we checked multicollinearity before entering these variables into the model. If evidence of multicollinearity was found (VIF > 10), the relevant variable was also deleted. Finally, the selected variables were classified into four broad groups in line with the multilevel analytic approach: health facility-, individuals-, household-, and community-level factors.

The health facility-level variables were general service readiness (facility’s management and infrastructure), LAMC services availability and readiness scores. For facilities that provide contraceptions, indices of severity of the availability of LAMC at the nearest health care facility and readiness were created following the WHO guidelines [37]. Seven variables were used to create contraception availability scores: combined oral contraceptive pills, progestin-only contraceptive pills, progestin-only injectable contraceptives, intrauterine device, implant, male sterilisation and female sterilisation. These indicator variables were classified in line with this study’s aim to generate LAMC availability scores: 1 point for each form of LAMC availability in the health facility and 0 for non-availability. Similarly, the contraceptive service readiness score was generated using seven dichotomous variables of healthcare personnel availability and training on providing LAMC. These scores were generated using the principal component analysis. The average distance on-road communication of the nearest LAMC-providing facility was also calculated and included as a health facility-level variable. Cluster’s LAMC-providing nearest health facility was identified first. Using Bangladesh road communication data, the average distance from the cluster to the nearest health facility was calculated separately for each of the eight administrative divisions. We used a regional-level average instead of the actual distance between the clusters and closest health facilities because the BHFS survey included a sample for all health facilities except the district health facility and maternal and child welfare centre. Thus, a cluster’s nearest health facility might not have been selected and included in the survey; hence, the actual distance would be problematic. The details of these computation procedures can be found elsewhere [19].

The individual-level variables are participants’ age (≤19 years, 20–34 years, ≥35 years), education (no formal education, primary, secondary, higher), and employment status (yes, no). The household-level variables are partners’ education (no formal education, primary, secondary, higher), partners’ occupation (agricultural worker, physical worker, service, business, others), household wealth quintile (poorest, poorer, middle, richer, richest) and the number of children ever given birth (no child, 1–2 children, > 2 children). BDHS generated wealth quintile variables using principal component analysis of the relevant data related to household assets. The scores generated were divided into five equal groups with a cut-off value of 0.20 and consecutively defined as poorest (0.20) to richest (1.00). Family types were also included as a household-level variable and classified as Nuclear family (if the family had < 5 members) and joint family (if the family had ≥5 members). The community-level factors were the place of residence (rural and urban) and administrative divisions.

### Statistical analysis

Descriptive statistics were used to summarise the characteristics of the respondents. Multilevel logistic regression was used to assess the associations of LAMC use with health facility-, individual- and household-, and community-level factors. The reason for using the multilevel regression was the hierarchical structure of the BDHS data, in which individuals are nested within a household and households are nested within a cluster. This created multiple dependencies in the data for which multilevel regression was deemed appropriate [38]. We followed the progressive model-building technique and developed four different models. Model 1 was the null model where no covariate was adjusted. Model 2 was the health facility-level model where LAMC use was considered with the health facility-level variables. Model 3 was the extension of Model 2 with the inclusion of individual- and household-level factors. All health facility-, individual-, households-, and community-level variables were adjusted in Model 4, which was the final model. Results were reported as adjusted Odds Ratios (aOR) with 95% Confidence Intervals (95% CI). The Intra-Class Correlation (ICC), Variance Inflation Factor (VIF), Akaike’s Information Criteria (AIC) and Bayesian Information Criteria (BIC) for each model were recorded and compared to select the best model. The ICC value was calculated by dividing the between-clusters-variance of LAMC use (random intercept variance) by the total variance of LAMC use (sum of between-clusters-variance and within-cluster (residual) variance of LAMC use). Statistical package R was used for analyses. All methods were performed in accordance with the relevant guidelines and regulations.

## Results

### Socio-economic characteristics of the respondents

Table 1 shows the background characteristics of the respondents. A majority of the respondents in this study were in 20–34 years age group (75%) at the time of the survey. Almost a quarter of women received no formal education, a third completed only primary and another third completed secondary education. Approximately 4% of women had no children and over 60% had 4 years or more intervals in their two most recent pregnancies. Around 74% of women resided in rural areas.

**Table 1:** Background characteristics of the respondents and distribution of LAMC, Bangladesh, 2017/18

	Overall	LAMC	Male sterelization	Female sterelization	IUD	Implants	Injectables
**Women’s age at birth of the last child**							
≤ 19 years	14.16	14.63	13.30	5.30	8.97	20.44	19.01
20–34 years	75.15	78.90	79.00	85.43	86.55	74.01	75.80
≥ 35 years	10.69	6.47	7.69	9.27	4.48	5.55	5.19
**Women’s education status**							
No formal education	24.05	23.21	40.30	30.03	22.42	18.89	18.61
Primary	35.45	39.63	45.43	39.30	29.82	38.90	39.96
Secondary	32.79	32.48	14.28	24.07	34.97	38.41	37.20
Higher	7.71	4.68	0.00	6.60	12.79	3.81	4.23
**Women’s employment status**							
Yes	52.24	59.80	67.98	54.29	49.84	60.49	60.89
No	47.76	40.20	32.02	45.71	50.16	39.51	39.11
**Partners’ education status**							
No formal education	28.32	33.39	43.17	36.95	26.96	31.96	31.17
Primary	32.64	37.37	39.32	31.29	32.38	41.36	38.98
Secondary	26.81	22.14	15.77	21.18	25.37	20.44	23.46
Higher	12.23	7.10	1.74	10.54	15.29	6.24	6.20
**Partners’ occupation**							
Agriculture work^a^	25.74	33.65	31.47	37.28	28.35	33.19	31.83
Physical labour^b^	40.59	42.36	48.05	36.30	38.79	43.13	45.22
Service	3.32	2.39	2.96	3.42	2.96	1.44	2.00
Business	16.17	19.89	13.50	19.66	26.96	21.68	0.14
Other	13.93	1.60	4.02	3.08	2.94	0.57	0.75
**Parity**							
No children	3.72	0.02	1.02	0.00	0.00	0.00	0.21
1–2 children	42.26	42.11	30.59	19.23	45.88	54.67	52.02
> 2 children	54.02	57.75	68.40	80.77	54.12	45.33	47.77
**Intervals between the two most recent live births**							
≤ 2 years	12.96	13.55	15.74	18.87	13.57	10.61	11.51
3–4 years	26.42	29.12	41.03	33.01	22.53	24.96	27.09
> 4 years	60.62	57.33	43.23	48.12	63.90	64.43	61.40
**Family type**							
Nuclear family^c^ (< 5 family members)	44.16	42.83	47.15	36.29	38.36	41.96	45.77
Joint family^d^ (≥5 family members)	54.84	57.17	52.85	63.71	61.64	58.04	54.23
**Household wealth status**^e^							
Poorest	20.44	26.60	32.22	20.48	16.37	34.51	27.84
Poorer	21.22	23.54	28.90	20.30	13.19	26.95	24.05
Middle	20.56	19.76	17.76	20.85	26.36	20.25	19.22
Richer	19.61	17.92	13.54	20.65	23.16	13.01	17.74
Richest	18.17	12.18	7.59	17.72	20.91	5.28	11.15
**Place of residence**							
Urban	25.92	25.77	22.09	26.46	35.39	20.46	26.38
Rural	74.08	74.23	77.91	73.54	64.61	79.54	73.62
**Region (administrative division)**							
Barishal	5.98	5.64	7.38	1.92	4.02	5.92	7.20
Chattogram	18.74	15.52	8.65	14.15	20.19	10.90	18.07
Dhaka	23.86	23.32	20.55	26.92	35.01	20.51	21.50
Khulna	12.50	12.63	10.45	12.38	15.28	10.70	12.88
Mymensingh	6.73	7.02	10.1	5.33	6.01	8.25	7.40
Rajshahi	14.01	15.61	11.01	18.24	12.58	17.44	14.76
Rangpur	12.43	16.42	28.90	14.78	2.92	22.59	15.28
Sylhet	5.76	3.83	2.96	6.28	3.99	3.68	2.92

^a^Farmer, fisherman, poultry rising, cattle rising, and home-based manufacturer

^b^Rickshaw driver, brick field worker, domestic servant, non-agricultural worker, carpenter, and masson

^c^ < 5 family members

^d^ ≥ 5 family members

^e^Classified based on the score generated from household’s assets by using principle component analysis. Details are provided in the explanatory variable section

### Distribution of long-acting modern contraceptives in Bangladesh

The distribution of long-acting modern contraceptives from health facilities in Bangladesh is presented in Table 1. We analysed data of 10,384 women, 3483 (34%) of them used LAMC. Injectable was the dominant LAMC, used by 55% of the overall LAMC users. Female sterilisation (25%), male sterilisation (6%), and implants (11%) were the other three major forms used by over 42% of LAMC users.

### Distribution of long-acting modern contraception use across women’s socio-economic characteristics

The overall and method-wise distribution of LAMCs are also presented in Table 1. Of the women who used LAMC, a majority of them were 20–34 years old (79%) and received either primary (40%) or secondary education (32%). Women engaged in formal income-generating activities (60%) were more likely than others to report using LAMC. Around 58% of the LAMC users had more than two children during the survey. The prevalence of LAMC users was higher among women in the Dhaka division (23%) than Rangpur and Chattogram divisions (16%). A similar pattern was recorded for the method-wise distribution of LAMCs. Male and female sterilisations were more common among people with two or more children, compared to IUDs, implants and injectables. Similarly, sterilisations were higher among those who reported having > 4 years of intervals between the two most recent live births (Table 1).

### Distribution of health facilities in Bangladesh

The distribution of health facilities across divisions is presented in Table 2. Of the 1524 health facilities, 1357 (89%) provided LAMC, and 93% (1262/1357) of these facilities were government hospitals/clinics located at the Upazila (i.e., sub-district) or in communities. Relatively high proportions of these facilities were located in the Dhaka (*n* = 245) and Chattogram (*n* = 234) divisions. Sylhet division had a lower number of health facilities with only 87 Upazila/community level governmental hospitals. NGO clinics (*n* = 19) and private hospitals (*n* = 9) were found highly clustered in the Dhaka division. The average distance between the health facilities and the BDHS clusters was around 6 km, higher in the Sylhet division (8 km) and lower in the Dhaka division (5 km). The mean score for the health facility management system was 0.79, higher in the Dhaka division (0.93) than in Khulna (0.87) and Chattogram (0.82) division. Similarly, higher availability of different LAMC was reported for Dhaka (mean score, 0.91) and Chattogram division (mean score, 0.74) than the corresponding average score of 0.69. Of the facilities where LAMCs were found available, 69% of them were functional and ready to provide LAMCs to the respondents. A higher score was reported for the healthcare facility of the Rangpur division (0.88) than for the Dhaka (0.85) and Chattogram (0.86) divisions.

**Table 2:** Division-wise distribution of health facilities, long-acting modern contraception (LAMC) availability, readiness to provide long-acting modern contraception, and health average distance from the demographic and health survey programme clusters in Bangladesh

Division	Availability of LAMC (***N*** = 1524)		Types of health facilities where LAMC is available (***N*** = 1357)					Average distance between home and health facility (km)	Health facility management	Health facility infrastructure	Severity of the availability of LAMC at the nearest health care facility	Health care facility readiness to provide LAMC
	Yes (***n*** = 1357)	No (***n*** = 167)	District hospital (***n*** = 4)	Upazila to community-level government hospital (***n*** = 1271)	Mother and Child Welfare Centre (***n*** = 7)	NGO clinic or hospital (***n*** = 55)	Private hospital (***n*** = 21)					
Barishal	106 (93.71)	7 (6.29)	1 (0.36)	101 (95.88)	1 (0.65)	3 (2.73)	0 (0.00)	6.43	0.75	0.76	0.70	0.47
Chattogram	261 (90.49)	27 (9.51)	1 (0.32)	245 (93.86)	1 (0.53)	9 (3.52)	5 (1.76)	5.85	0.82	0.82	0.74	0.86
Dhaka	264 (87.07)	39 (12.93)	1 (0.32)	234 (88.63)	1 (0.41)	19 (7.30)	9 (3.34)	4.83	0.93	0.97	0.91	0.85
Khulna	175 (93.21)	13 (6.79)	1 (0.31)	165 (94.41)	1 (0.62)	7 (4.19)	1 (0.48)	5.92	0.87	0.92	0.63	0.57
Rajshahi	201 (91.54)	19 (8.46)	0 (0.00)	191 (94.75)	1 (0.50)	7 (3.32)	3 (1.24)	7.12	0.74	0.81	0.58	0.67
Rangpur	147 (76.36)	46 (23.64)	0 (0.00)	141 (95.59)	1 (0.63)	3 (2.24)	2 (1.45)	5.98	0.85	0.89	0.72	0.88
Sylhet	93 (96.13)	4 (3.87)	0 (0.00)	87 (94.22)	1 (0.50)	4 (3.54)	1 (1.41)	8.34	0.67	0.65	0.64	0.43
Mymensingh	110 (90.0)	12 (10.0)	0 (0.00)	106 (96.43)	0 (0.00)	3 (2.66)	1 (0.88)	6.44	0.69	0.78	0.62	0.52
**Grand average distance**								**6.36**	**0.79**	**0.83**	**0.69**	**0.65**

### Model selection

The associations of LAMC use with health facility-, individual-, household-, and community-level variables were assessed through multilevel logistic regression models. Of the four models ran separately, the AIC, BIC and ICC values were compared to identify the best models. The preferred model was the one that had the smallest AIC, BIC and ICC (Table 3). According to these markers, Model 4 (including health facility-, individual-, household-, and community-variables) fitted the data better. The ICC value for the null model (Model 1) suggested around 17% differences in LAMC use across clusters included in the BDHS. However, this value was reduced to only 3% once health facility-, individual-, household-, and community-level factors were included in the final model. Around 11% of such reduction was reported once health facility-level factors were included in the null model.

**Table 3:** Multilevel logistic regression models assessing the relationships between the use of long-acting modern contraception and health facility-, individual-, household-, and community-level factors (*N* = 10,384)

Characteristics	Null model	Health facility-level model, aOR (95% CI)	Health facility-, individual-, and household-level model, aOR (95% CI)	Health facility-, individual-, household-, and community-level model, aOR (95% CI)
**General health service readiness**				
Health facility management system		1.76 (1.18–2.36)^**^	1.66 (1.28–2.40)^**^	1.59 (1.21–2.42)^*^
Health facility infrastructure		1.48 (0.98–1.78)	1.47 (0.90–1.95)	1.44 (1.01–1.69)^**^
**Degree of availability of LAMCs at the nearest health care facility**		2.17 (1.19–3.20)^**^	2.23 (1.43–3.76)^*^	2.16 (1.18–3.21)^*^
**Readiness of the nearest health care facility (where LAMCs are available) to provide LAMCs**		1.76 (1.14–3.18)^**^	1.64 (1.16–3.20)^**^	1.74 (1.15–3.20)^**^
**Average distance on road communication from women’s resided cluster to the nearest health facility where LAMCs are available**		0.78 (0.44–0.98)^**^	0.74 (0.42–0.97)^**^	0.73 (0.46–0.96)^**^
**Women’s age**				
≤ 19 (ref)			1.00	1.00
20–34			0.82 (0.71–0.96)^**^	0.84 (0.72–0.97)^**^
≥ 35			0.61 (0.48–0.78)^**^	0.63 (0.50–0.80)^**^
**Women’s education status**				
No formal education (Ref)			1.00	1.00
Primary			1.52 (1.33–1.73)^**^	1.55 (1.36–1.77)^**^
Secondary			1.73 (1.48–2.03)^**^	1.79 (1.53–2.10)^**^
Higher			1.33 (1.02–1.74)^**^	1.34 (1.03–1.75)^**^
**Women’s employment status**				
No (ref)			1.00	1.00
Yes			1.41 (1.27–1.57)^**^	1.37 (1.23–1.52)^**^
**Husbands’ education status**				
No education (ref)			1.00	1.00
Primary			0.91 (0.81–1.04)	0.93 (0.82–1.05)
Secondary			0.60 (0.52–0.69)^**^	1.61 (1.53–1.71)^**^
Higher			0.43 (0.34–0.55)^**^	1.44 (1.34–1.55)^**^
**Husbands’ occupation**				
Agricultural worker (ref)			1.00	1.00
Physical worker			0.92 (0.82–1.04)	0.88 (0.78–0.99)^*^
Services			1.24 (0.91–1.90)	1.18 (0.86–1.61)
Business			1.29 (1.11–1.48)^**^	1.23 (1.06–1.42)^**^
Other			0.26 (0.07–0.90)^**^	0.26 (0.08–0.91)^**^
**Number of children ever given birth**				
No children (ref)			1.00	1.00
1–2 children			9.93 (4.70–20.96)^*^	9.84 (4.66–20.81)^*^
> 2 children			9.90 (4.70–20.85)^*^	9.92 (4.71–20.91)^*^
**Intervals between the two most recent live births**				
≤ 2 years			1.00	1.00
3–4 years			1.07 (0.91–1.25)	1.06 (0.90–1.24)
> 4 years			0.97 (0.84–1.13)	0.95 (0.81–1.10)
**Family type**				
Nuclear (ref)			1.00	1.00
Joint			1.18 (1.06–1.30)^**^	1.21 (1.10–1.35)^**^
**Household wealth quintile**				
Richest (ref)			1.00	1.00
Richer			1.32 (1.11–1.57)^**^	1.41 (1.18–1.68)^**^
Middle			1.52 (1.27–1.82)^**^	1.71 (1.42–2.06)^**^
Poorer			1.81 (1.50–2.18)^**^	2.09 (1.72–2.54)^**^
Poorest			2.54 (2.09–3.10)^**^	2.94 (2.38–3.62)^**^
**Place of residence**				
Urban (ref)				1.00
Rural				0.66 (0.57–0.77)^**^
**Region (administrative division)**				
Barishal (ref)				1.00
Chottogram				1.11 (0.85–1.43)
Dhaka				1.46 (1.13–1.89)^**^
Khulna				1.18 (0.91–1.53)
Mymensingh				1.16 (0.88–1.53)
Rajshahi				1.44 (1.11–1.87)^**^
Rangpur				1.59 (1.22–2.06)^**^
Sylhet				0.77 (0.58–1.02)
**Model summary**				
AIC	12,991.51	11,146.06	10,969.60	10.912.18
BIC	13,006	11,325.92	11,139.61	11,138.87
ICC	0.17	0.11	0.10	0.07
Median Odds Ratio	1.80	1.78	1.78	1.68
Variance of the random intercept	0.38 (0.04)	0.45 (0.03)	0.61 (0.04)	0.55 (0.404)

*ref* Reference group, ^**^p < 0.01, ^*^p < 0.05

### Factors associated with long-acting modern contraception use

In the final model, we found that health facility-level factors were strong predictors of LAMC use following the adjustment of health facility-, individual-, household-, and community-level factors (Table 3). Women were 1.59 times (95% CI, 1.21–2.42) and 1.44 times (95% CI, 1.01–1.69) likely to report using LAMCs for every unit increase in the score of health facility management and health facility infrastructure of the nearest health facility, respectively. The likelihood of LAMCs used increased by 2.16 times (95% CI, 1.18–3.21) for each unit increase in the degree of availability of LAMCs at the nearest healthcare facility. Similarly, for every unit increase in the readiness of the nearest health care facility (where LAMCs are available) to provide LAMCs, the aOR increased to 1.74 (95% CI, 1.15–3.20). The likelihood of women using LAMCs was reduced by 27% (aOR, 0.73; 95% CI, 0.46–0.96) for every km increase in the average distance on road communication of health facility from respondents’ resided cluster.

At the individual level, lower likelihoods of LAMCs use were found among women aged 20–34 years (aOR, 0.83; 95% CI, 0.74–0.93) and ≥ 35 years (aOR, 0.56; 95% CI, 0.46–0.67) as compared to the women aged ≤19 years. Women engaged in formal employments and with increased years of education were more likely to report using LAMC than women who received no formal education and were not employed in a formal job.

Women who received primary, secondary, or further education and whose partners received secondary or higher education were significantly more likely than others to use LAMCs. Women living in a joint family were more likely to use LAMCs than women living in a nuclear family (aOR, 1.21; 95% CI, 1.10–1.35). LAMCs use was around 9.84 times (95% CI, 4.66–20.81) and 9.92 times (95% CI, 4.71–20.91) higher among women with 1–2 children and > 2 children, respectively, compared to women who had no children. Furthermore, LAMCs utilisation was found to be higher among women with an increasing level of household wealth.

The likelihood of LAMC use was around 41% lower (95% CI, 0.61–0.77) among rural women than in urban women. Women in Dhaka (aOR, 1.46; 95% CI, 1.13–1.89), Rajshahi (aOR, 1.44; 95% CI, 1.11–1.87), and Rangpur (aOR, 1.59; 95% CI, 1.22–2.06) divisions were more likely to use LAMC than the women in the Barishal division.

### Effects of health facility environment on long-acting modern contraception use

We calculated the health facility environment by considering all health facilities providing LAMC in 6.3 km (division level average distance on road-communication of health facility from the BDHS cluster) buffer distance. The number of health facilities and their characteristics were generated for national and urban and rural locations (Table 4). The likelihood of LAMC use was twofold among women living in areas where there was a health facility within 6.3 km (aOR 2.01; 95% CI 1.67–2.37). The likelihood increased further (aOR 3.39; 95% CI 1.84–4.90) if the 6.3 km buffer distance had more than one health facility compared to having no health facility. These likelihoods increased even further for rural areas. The general health services readiness, degree of availability of LAMCs at the nearest health care facility, and readiness of the nearest health care facility (where LAMCs are available) to provide LAMCs were also found significantly associated with LAMC use (Table 4).

**Table 4:** Multilevel logistics regression assessing the relationships between of LAMC use and attributes of health facilities located within 6.36 km distance from the BDHS clusters

Health facility characteristics	Long-acting modern contraception use, aOR (95% CI)		
	Overall^a^	Rural^a^	Urban^a^
**Number of health facilities**			
0	1.00	1.00	–
1	2.01 (1.67–2.37)^**^	3.65 (1.97–4.38)^**^	1.00
≥ 2	3.39 (1.84–4.90)^*^	4.87 (2.12–5.34)^*^	2.73 (1.73–3.57)^**^
**General health service readiness**			
Health facility management system	2.47 (1.13–2.29)^**^	3.16 (1.51–4.43)^**^	1.66 (1.28–2.40)^**^
Health facility infrastructure	1.56 (0.98–1.80)	1.48 (1.01–1.78)^**^	1.47 (0.90–1.95)
**Degree of availability of LAMCs at the nearest health care facility**	2.21 (1.12–3.23)^**^	1.19 (0.78–2.13)	3.02 (1.43–4.13)^**^
**Readiness of the nearest health care facility (where LAMCs are available) to provide LAMCs**	1.38 (1.23–3.40)^**^	1.06 (0.84–1.98)	1.64 (1.16–3.20)^**^

^a^Models adjusted with women’s age, education, employment status, number of children ever given birth, Intervals between the two most recent live births, husbands’ education, occupation, family type, household wealth quintile, place of residence and region

^**^p < 0.01, ^*^p < 0.05

We also conducted a sub-group analysis to explore the association between health facility level factors with permanent modern methods and long-acting reversible methods used (results now shown in the table). The observed associations were found consistent with the observations reported in Tables 3 and 4. However, the associations were stronger for permanent than reversible methods. For instance, aOR between the degree of availability of LAMCs at the nearest health care facility was and women’s using permanent methods was 2.38 (95%CI, 1.70–3.08) compared to 3.98 (95%CI 1.34–2.62) for reversible methods (results now shown in the table).

## Discussion

This study provides new insights for LMICs and Bangladesh on the role of availability and accessibility of health facilities in determining LAMC use. Only 34% of the total respondents analysed used LAMC. LAMC methods are available in 89% of the health facilities in Bangladesh; however, 65% of them are found functionally ready to provide these services. The findings suggest the average distance of a health facility that provides LAMC from the respondents’ homes was 6.36 km, highest in Sylhet division (8 km) and lowest in Dhaka division (5 km). The LAMC uptake was positively associated with LAMC availability at the nearest health facility and improved management and infrastructure of those facilities. These relationships were stronger in rural than urban areas. The number of health facilities providing LAMC within 6.3 km was revealed as the strongest predictor of LAMC use. Women from lower socio-economic backgrounds were more likely than women of other wealth groups to use LAMC. Overall, our findings suggest that LAMC uptake would increase among women if there are available LAMC-providing facilities with good infrastructure in proximity to women’s homes.

Although the SDGs target to ensure universal coverage of sexual and reproductive healthcare services by 2030 [39], the Bangladesh government has set the target to achieve 75% coverage of modern contraceptive use by 2025 [40, 41]. Achieving this target is deemed a real challenge as only 54% of the eligible couples in Bangladesh use modern contraception [27], and this percentage is even lower for LAMCs (34%), as found in this study. This estimated prevalence of LAMCs in Bangladesh is lower than the global (47.6%) and LMICs prevalences (47.0%) but higher than in the African region (19%) [42]. The prevalence of LAMCs was also reported higher in two neighbouring countries, India (41%) and Myanmar (38%) [15].

In Bangladesh, the programmes for improving health facility management, infrastructure and accessibility to LAMCs were first taken into account as part of the 7th Five-Year Plan (2016–2020) [43] — and are currently functional as part of the 8th Five-Year Plan (2021–2026) [44]. However, no notable progress has been observed to date. Providing contraception services, including LAMC, via family planning workers (who visit couples’ homes fortnightly) is still the fundamental way to provide family planning services and contraception at the community level [27]. An advantage of this approach is that couples who are not interested in accessing services from health facilities can still access contraception services. However, this approach has two major disadvantages: i) family planning workers usually lack specialised medical education and ii) family planning workers are restricted to provide non-surgical contraception methods, mainly pills and condoms [2]. Consequently, pills (25%) and condoms (7%) are the main contraceptives in Bangladesh and are used by 60% of the overall contraception users [27]. This pattern differs from many other LMICs where condoms and female sterilisation are the dominant contraception methods [13] These indicate the dominant role of family planning workers in providing contraception services over health facilities [2, 28]. Furthermore, the failure rates of pills (5–15%) and condoms (7–19%) are very high in their typical use [46]. Therefore, the ongoing provision of contraception services via family planning workers needs to be improved to reduce unintended pregnancies and related adverse consequences [2] as well as to achieve SDGs targets to reduce maternal and child mortality.

Although several programs to increase LAMC use have been introduced in Bangladesh, including payment for sterilisation or vasectomy, progress is unlikely to be significant unless the provider-level challenges are addressed [47, 48]. The first challenge comes from the lack of coordination between family planning workers and health facilities. In Bangladesh, family planning workers work under the Directorate General of Family Planning Services, and health facilities function under the Directorate General of Health Services [49]. One of the job responsibilities of family planning workers is promoting awareness about LAMC use among women in rural areas. They educate eligible couples on family planning methods and contraception use. They do not provide LAMC to interested people but refer them to health facilities [50]. As a result, the coordination between family planning workers and health facilities is minimal [2]. Although family planning workers have direct contact with eligible couples, they cannot monitor whether they accessed health facilities for LAMC or not. Such a lack of coordination results in unequal coverage and a significant pathway of miss-out from the services [13]. Effective linkages between family planning workers and health facilities are crucial. However, because of family planning workers’ direct access to the grassroots level, LAMC use among disadvantaged groups and in rural Bangladesh is relatively high, which is unique to the results of studies conducted in LMICs, including Bangladesh [16, 17, 32, 33]. However, LAMC use is still relatively low, even among the women of the poorest and poorer wealth quintiles.

The second challenge comes from the health facility. Because of the government’s efforts, LAMC is available in most healthcare facilities in Bangladesh. As per the results of this study, it is available in 89% of healthcare facilities. However, 69% of them are functionally ready to provide LAMC with high divisional variations. Over 85% of health facilities in Rangpur, Dhaka, and Chattogram divisions had functional readiness to provide LAMC. Nearly half of the healthcare facilities in the remaining five divisions were not functionally ready to provide LAMC, although services were available there. This pattern has created several challenges at the field level, including the crowdedness in the nearest LAMC-providing healthcare facility and increased distance between women’s homes and that facility.

Availability of LAMC-providing health facilities in short distances has been identified as a critical predictor of LAMC uptake in this study, a consistent finding in other LMICs such as Turkey and Zambia [34, 36, 51]. This finding also indicates that some people may not access LAMC due to the relatively high distance of the LAMC-providing facilities despite having an intention. There may have a compound effect of this disadvantage among women with low educational achievement and relatively low knowledge of LAMC. Social barriers and taboos, a low agency of decision making and financial inability can prevent less educated women from visiting health facilities to access LAMC if located far away [2]. However, this understanding may not be true for younger women, who are comparatively educated and enjoy more autonomy than older women. Moreover, limited and distant LAMC-providing healthcare facilities are often overcrowded. Furthermore, some healthcare facilities are not women-friendly; for instance, they do not have private space to discuss LAMC use [2]. These challenges create inequality in LAMC use, wherein LAMC-providing healthcare facilities (> 93% are government owned) tend to serve relatively poor people. On the other hand, educated and wealthy people choose private clinics to access LAMC (which are limited in Bangladesh) or use short-acting methods, including condoms or pills from private sources. These observations highlight the requirement for more healthcare facilities and infrastructural development of the existing healthcare facilities that can provide LAMC at the community level. Engaging 13,221 community clinics that are currently functioning could accelerate the initiative.

Our findings also suggest improvement in the management of health facilities has significant contributions to the increased use of LAMC. In Bangladesh, there is a significant shortage of trained healthcare professionals assigned to LAMC and other contraception services [52]. Moreover, healthcare providers are expected to provide counselling in conjunction with LAMC [44]. However, many practitioners, particularly those who completed a Bachelor of Medicine or Bachelor of Surgery degree, may not prioritise counselling over other competing tasks. Moreover, in most healthcare facilities, a substantial proportion of healthcare providers are male, and women usually do not feel comfortable accessing contraception services through a male provider [52]. These challenges are even deep-rooted in rural health facilities along with poor management, including inadequate medical supplies, inability to use modern technologies in providing health services and absence of assigned healthcare personnel [53]. Consequently, women in rural areas are less likely to use LAMC.

This study has several limitations. Data analysed in this study were extracted from cross-sectional surveys. Therefore, the findings are correlational only, not casual. To secure the privacy of the respondents, BDHS displaced clusters’ locations up 0–5 km for rural areas and 0–2 km for urban areas. Therefore, the calculated average distance of the nearest health facility could be slightly different from the actual distance. However, the BDHS ensured that the new perturbed locations fell within the designated administrative boundaries. Therefore, errors from displacement are likely to be random and minimum. A previous study found that the effect of this variation is insignificant [54]. Moreover, the contraceptive methods that were included to generate LAMC variables widely vary from one another, and their seeking processes are also different. For instance, the female sterilisation methodd need to be performed in -a well-developed in-patient settings by expert healthcare personnel immediately following childbirth. On the contrary, injectable methods are comparatively easy, do not need a high-end in-patient settings and highly skilled healthcare personnel, and can be applied at any time. These disparities may limit the interpretability of this study’s findings. However, we have conducted a sub-group analysis to determine the association of health facility level factors on permanent modern and long-acting reversible modern methods. The association reported were consistent with our main study findings. This confirms that the findings of this study are still valid. Regardless of these limitations, to our knowledge, this is the first study in the context of LMICs that examined the influence of health facility-level factors adjusted with the individual-, household-, and community-level factors. Clusters of LAMC use and non-use were identified, and the factors associated with LAMC were determined using multilevel regressions with adjustments for a wide range of factors. Therefore, the finding of this study is likely to be precise and can be generalisable in countries with similar features to Bangladesh and may help develop evidence-based policies.

## Conclusion

During 2017–2018, around 33% of women used LAMC in Bangladesh. Health facility-level factors, including health facility infrastructure, management, and readiness to provide LAMC were strong predictors of LAMC use in Bangladesh and the effects of the predictors were substantial in rural than urban areas. Health facilities providing LAMC located at a closer distance were positively associated with increased uptake of LAMC. Health facilities should be strengthened to provide LAMC and more health facilities of such capacities are needed. The current provision of family planning services through the field-level workers also needs to be strengthened with strong coordination and referral linkages with the health facilities.

## Data Availability

The data that support the findings of this study are available from The DHS Program, but restrictions apply to the availability of these data, which were used under license for the current study, and so are not publicly available. Data are, however, available from the authors upon reasonable request and with permission of The DHS Program.

## Abbreviations

LAMC: Long-acting modern contraception
SDG: Sustainable Development Golas
LMICs: Low- and middle-income countries
BDHS: Bangladesh Demographic and Health Survey
BHFS: Bangladesh Health Facility Survey
MCWC: Mother and Child Welfare Centre
FPW: Family Planning Worker
aOR: Adjusted Odds Ratios
AIC: Akaike Information Criteria
BIC: Bayesian Information Criteria

## References

[1] Bearak J, Popinchalk A, Ganatra B, Moller A-B, Tunçalp Ö, Beavin C, Kwok L, Alkema L (2020). Unintended pregnancy and abortion by income, region, and the legal status of abortion: estimates from a comprehensive model for 1990–2019. Lancet Glob Health.

[2] Khan MN, Harris M, Loxton D (2020). Modern contraceptive use following an unplanned birth in bangladesh: an analysis of national survey data. Int Perspect Sex Reprod Health.

[3] Goossens J, Van Den Branden Y, Van der Sluys L, Delbaere I, Van Hecke A, Verhaeghe S, et al. The prevalence of unplanned pregnancy ending in birth, associated factors, and health outcomes. Hum Reprod. 2016;31(12):2821–33. 10.1093/humrep/dew266 27798048

[4] World Health Organization (2015). Contraception.

[5] Khan MN, Harris ML, Loxton D (2020). Assessing the effect of pregnancy intention at conception on the continuum of care in maternal healthcare services use in Bangladesh: evidence from a nationally representative cross-sectional survey. Plos One.

[6] Khan MN, Harris ML, Shifti DM, Laar AS, Loxton D (2019). Effects of unintended pregnancy on maternal healthcare services utilization in low-and lower-middle-income countries: systematic review and meta-analysis. Int J Public Health.

[7] Qiu X, Zhang S, Sun X, Li H, Wang D (2020). Unintended pregnancy and postpartum depression: a meta-analysis of cohort and case-control studies. J Psychosom Res.

[8] Darroch JE, Singh S, Nadeau J. Contraception: an investment in lives, health and development. Gutmacher Institute & UNFPA. 2008;5:1–4. 19537329

[9] Shukla A, Kumar A, Mozumdar A, Aruldas K, Acharya R, Ram F, Saggurti N (2020). Association between modern contraceptive use and child mortality in India: a calendar data analysis of the National Family Health Survey (2015-16). SSM Popul Health.

[10] Shoupe D (2016). LARC methods: entering a new age of contraception and reproductive health. Contracept Reprod Med.

[11] Assembly G (2030). Sustainable Development Goals. Transform Our World 2015.

[12] Khan MN. Effects of unintended pregnancy on maternal healthcare services use in Bangladesh. Faculty of Health and Medicine, School of Medicine and Public Health, The University of Newcastle, Australia; 2021.

[13] Khan MN, Islam MM (2022). Pregnancy resulting from contraceptive failure is increasing in Bangladesh: evidence from nationally representative demographic and health survey. Sci Rep.

[14] Government of the People’s Republic of Bangladesh (2011). The Sixth-Five Year Plan, 2011–2016.

[15] Blumenberg C, Hellwig F, Ewerling F, Barros AJ (2020). Socio-demographic and economic inequalities in modern contraception in 11 low-and middle-income countries: an analysis of the PMA2020 surveys. Reprod Health.

[16] Apanga PA, Kumbeni MT, Ayamga EA, Ulanja MB, Akparibo R (2020). Prevalence and factors associated with modern contraceptive use among women of reproductive age in 20 African countries: a large population-based study. BMJ Open.

[17] Lasong J, Zhang Y, Gebremedhin SA, Opoku S, Abaidoo CS, Mkandawire T, Zhao K, Zhang H (2020). Determinants of modern contraceptive use among married women of reproductive age: a cross-sectional study in rural Zambia. BMJ Open.

[18] Ahinkorah BO, Seidu A-A, Appiah F, Budu E, Adu C, Aderoju YBG, Adoboi F, Ajayi AI (2020). Individual and community-level factors associated with modern contraceptive use among adolescent girls and young women in Mali: a mixed effects multilevel analysis of the 2018 Mali demographic and health survey. Contracept Reprod Med.

[19] Tegegne TK, Chojenta C, Forder PM, Getachew T, Smith R, Loxton D (2020). Spatial variations and associated factors of modern contraceptive use in Ethiopia: a spatial and multilevel analysis. BMJ Open.

[20] Hong R, Montana L, Mishra V (2006). Family planning services quality as a determinant of use of IUD in Egypt. BMC Health Serv Res.

[21] Wang W (2012). How family planning supply and the service environment affect contraceptive use: findings from four East African countries.

[22] Rose M, Abderrahim N, Stanton C, Helsel D. Maternity care. A comparative report on the availability and use of maternity services. Measure Evaluation Technical Report Series, No. 9. 1999.

[23] Hossain M, Khan M, Ababneh F, Shaw J (2018). Identifying factors influencing contraceptive use in Bangladesh: evidence from BDHS 2014 data. BMC Public Health.

[24] Haq I, Sakib S, Talukder A (2017). Sociodemographic factors on contraceptive use among ever-married women of reproductive age: evidence from three demographic and health surveys in Bangladesh. Med Sci.

[25] Skiles MP, Burgert CR, Curtis SL, Spencer J (2013). Geographically linking population and facility surveys: methodological considerations. Popul Health Metrics.

[26] Household L (2014). DHS Spatial Analysis Reports 10.

[27] National Institute of Population Research and Training (NIPORT) (2020). aI: Bangladesh Demographic and Health Survey 2017–18.

[28] 2019 NIoPRaTNaI (2019). Bangladesh Health Facility Survey 2017.

[29] Anguzu R, Sempeera H, Sekandi JN (2018). High parity predicts use of long-acting reversible contraceptives in the extended postpartum period among women in rural Uganda. Contracept Reprod Med.

[30] Bhandari R, Pokhrel KN, Gabrielle N, Amatya A (2019). Long acting reversible contraception use and associated factors among married women of reproductive age in Nepal. Plos One.

[31] Bintabara D, Nakamura K, Seino K (2017). Determinants of facility readiness for integration of family planning with HIV testing and counseling services: evidence from the Tanzania service provision assessment survey, 2014–2015. BMC Health Serv Res.

[32] Cahill N, Sonneveldt E, Stover J, Weinberger M, Williamson J, Wei C, Brown W, Alkema L (2018). Modern contraceptive use, unmet need, and demand satisfied among women of reproductive age who are married or in a union in the focus countries of the Family Planning 2020 initiative: a systematic analysis using the Family Planning Estimation Tool. Lancet.

[33] Huda FA, Robertson Y, Chowdhuri S, Sarker BK, Reichenbach L, Somrongthong R (2017). Contraceptive practices among married women of reproductive age in Bangladesh: a review of the evidence. Reprod Health.

[34] Sato R, Rohr J, Huber-Krum S, Esmer Y, Okçuoğlu BA, Karadon D, et al. Effect of distance to health facilities and access to contraceptive services among urban Turkish women. Eur J Contracept Reprod Health Care. 2021;26(5):374–82. 10.1080/13625187.2021.1906412 33874821

[35] Shiferaw S, Spigt M, Seme A, Amogne A, Skrøvseth S, Desta S, Radloff S, Tsui A, GeertJan D (2017). Does proximity of women to facilities with better choice of contraceptives affect their contraceptive utilization in rural Ethiopia?. Plos One.

[36] Wang W, Mallick L (2019). Understanding the relationship between family planning method choices and modern contraceptive use: an analysis of geographically linked population and health facilities data in Haiti. BMJ Glob Health.

[37] Organization WH (2015). Service availability and readiness assessment (SARA).

[38] Diez-Roux AV (2000). Multilevel analysis in public health research. Annu Rev Public Health.

[39] UN General Assembly (2015). Transforming our world : the 2030 Agenda for Sustainable Development.

[40] Bangladesh Planning Commission (2021). General Economic Division: Eighth Five Year Plan (FY2020-FY2025): Promoting Posperity and Fostering Inclusiveness.

[41] Planning Commission (2016). General Economic Division: Seventh Five Year Plan (FY2016–FY2020): Accelerating Growth, Empowering Citizens.

[42] United Nations (2015). Trends in Contraceptive Use Worldwide 2015.

[43] Government of the People’s Republic of Bangladesh (2016). 7th Five Year Plan, FY 2016-FY2020.

[44] Government of the People’s Republic of Bangladesh (2021). 8th Five Year Plan, FY 2021-FY2026.

[45] Schuler SR, Hashemi SM, Jenkins AH. Bangladesh's family planning success story: a gender perspective. Int Fam Plan Perspect. 1995;6:132–66.

[46] Polis C, Bradley SE, Bankole A, Onda T, Croft TN, Singh S (2016). Contraceptive failure rates in the developing world: an analysis of demographic and health survey data in 43 countries.

[47] Donahoe DA. Men and family planning in Bangladesh: a review of the literature. Population Council. 1996.

[48] Cleland J, Mauldin WP (1991). The promotion of family planning by financial payments: the case of Bangladesh. Stud Fam Plan.

[49] Organization WH (2015). Bangladesh health system review.

[50] Das TR (2016). Family planning program of Bangladesh: achievements and challenges. South East Asia J Public Health.

[51] Silumbwe A, Nkole T, Munakampe MN, Milford C, Cordero JP, Kriel Y, Zulu JM, Steyn PS (2018). Community and health systems barriers and enablers to family planning and contraceptive services provision and use in Kabwe District, Zambia. BMC Health Serv Res.

[52] Khan MN, Islam, M Mofizul. Women's experience of unintended pregnancy and changes in contraceptive methods: evidence from a nationally representative survey. Reprod Health. 2022;19(1):187. 10.1186/s12978-022-01492-w PMC9438238 36050768

[53] Hassan N, Rashid H, Das R. Smart Health Management System for Rural Area of Bangladesh Utilizing Smartphone and NID. Health care. 2017;1:3.

[54] Sato RKM, Elewonibi B, Mhande M, Msuya S, Shah I, Canning D (2019). Distance to facility and healthcare utilization in Tanzania: unbiased and consistent estimation using perturbed location data. Population Association of America.

